# Impact of the COVID-19 pandemic on the psychological aspects and mental health of elite soccer athletes: a systematic review

**DOI:** 10.3389/fpsyg.2023.1295652

**Published:** 2024-01-25

**Authors:** Alexandro Andrade, Anderson D'Oliveira, Henrique Pereira Neiva, Gilberto Gaertner, Whyllerton Mayron da Cruz

**Affiliations:** ^1^Health and Sports Science Center – CEFID/Santa Catarina State University–UDESC, Florianópolis, Santa Catarina, Brazil; ^2^Laboratory of Sports and Exercise Psychology–LAPE, Florianópolis, Santa Catarina, Brazil; ^3^Department of Sport Sciences, University of Beira Interior, Covilhã, Portugal; ^4^Research Center in Sports Sciences, Health Sciences and Human Development, CIDESD, Covilhã, Brazil

**Keywords:** athletes, anxiety, mood states, mental health, social isolation

## Abstract

**Introduction:**

Investigation of the psychological impact on soccer athletes during the pandemic is essential given their unique challenges, including training disruptions and competition postponements. Understanding these effects will allow the development of specific strategies to preserve the mental health and performance of elite athletes, contributing to effective interventions with both short and long-term benefits.

**Objective:**

To analyze the impact of the COVID-19 pandemic on the psychological aspects and mental health of elite soccer athletes.

**Method:**

The review adhered to PRISMA criteria, and the study protocol was registered in the International Prospective Register of Systematic Reviews (CRD42022341545). Searches were conducted until July 2023 in databases including Cochrane, PsycINFO, PubMed, Scopus, SPORTDiscus, and Web of Science. Only original, peer-reviewed studies in English, Portuguese, or Spanish assessing the impact of the COVID-19 pandemic on the psychological aspects and mental health of elite soccer athletes were included.

**Results:**

The search identified 1,055 records and 43 studies were included in this review between 2020 and 2023. In total, the sample included 16,321 soccer athletes of different age groups. Anxiety, depression, mood states, and mental well-being were the most investigated variables. Increased levels of anxiety, depression, and worsening mental well-being were observed in elite soccer athletes. Maintaining fitness during the pandemic showed positive results. Other variables, such as coping, resilience, and sleep quality monitoring, were less widely investigated. Evaluating methodological quality was considered regular for observational and experimental studies.

**Conclusion:**

The study reveals a negative impact of the COVID-19 pandemic on elite soccer athletes, considering psychological aspects and their mental health, notably heightened anxiety and depression. Observational methods predominated, showing mood swings linked to individual characteristics and fitness maintenance efforts. Studies with better-designed methodological approaches and controlled experimental interventions are recommended in the future to mitigate the negative effects of the pandemic on soccer players.

**Systematic review registration:**

https://www.crd.york.ac.uk/prospero/display_record.php?, identifier (CRD42022341545).

## Introduction

In 2020, the COVID-19 pandemic impacted the world in all areas and caused unprecedented extreme damage to the world sports calendar, a fact that had not occurred since World War 2 (IOC, 2020). In the absence of vaccines and antiviral drugs, non-pharmaceutical interventions implemented in response to virus damage were the only options available to slow and moderate the spread of the virus through the population (Kenyon, 2020; Mann et al., 2020). In addition to quarantine and isolation procedures for those who have been exposed to or infected with COVID-19, social distancing is applied as a strategy to reduce the transmission of COVID-19, with the practice of confinement and social isolation being the most effective and widely used (Haug et al., 2020).

Such factors have led to the need to study the impact of the COVID-19 pandemic on the psychological aspects and mental health of athletes. In addition, the complex psychosocial situation of confinement has caused important changes and serious damage to the quality and quantity of training of athletes, and these factors have brought many uncertainties about the future of the sports environment (Andreato et al., 2020). Factors such as the abrupt change in their daily routine, the home confinement measures adopted, and uncertainty about the date of return to activities can lead athletes to experience conditions that affect their mental health, including external sources of distress, anxiety, mood swings, and other concerns and tensions (Evli et al., 2020; Jansen, 2021; Romdhani et al., 2022).

The systematic reduction in training caused losses in physical performance capacity, which meant a loss of competitiveness on the return to competition, generating a cascade of losses that still need to be better understood. In addition to the physical and technical damage, the psychological impact is of great concern to the athlete. Thus, sports science professionals and scientists are challenged to help athletes deal with some of these relevant aspects in times of a pandemic. As a consequence, several recent recommendations suggest minimizing the negative impacts of the COVID-19 pandemic on athletes in general (Andreato et al., 2020; Girardi et al., 2020; Farooq et al., 2021) and in soccer (Jia et al., 2022). Predominantly, the athletes had to deal with the frustration of their goals and changes in their usual training routines (Zinner et al., 2020; Leguizamo et al., 2021).

Soccer arouses great interest in the scientific community, and given its importance in sports sciences and other areas, it is currently the most investigated sport modality (Kirkendall, 2020). A challenging situation is to establish a procedure with implications for their well-being and mental health, while at the same time enabling increased *performance* (Leguizamo et al., 2021). It is known that psychological factors predispose athletes and that changes in sports performance are common and should be considered in sports development and preparation programs (Dönmez et al., 2022). Recent studies have shown implications of the COVID-19 pandemic, with a negative impact on mental status during the pandemic, including concerns about the sporting future, which caused increased anxiety and stress, as well as worsened sleep quality in athletes of various modalities (Håkansson et al., 2020; Mon-López et al., 2020; Jurecka et al., 2021). To date, there are no systematic reviews on whether psychological interventions and approaches were used and if they were effective during the pandemic.

In this sense, the current systematic review with a specific focus on soccer, aims to form a knowledge base, providing information from the evidence with approaches based on sports psychology and analyzing the trends in the evidence. A recent review investigated the effect of the COVID-19 pandemic and its implications on the mental and emotional health of athletes in a generic way (Jia et al., 2022). The authors reported that this phenomenon is complex and multifaceted, as the lack of social interactions with coaches and teammates, the absence of continuous access to training facilities and direct contact with professionals, and the lack of active use of healthy coping mechanisms resulted in worsening mental health of athletes in the COVID-19 era (Jia et al., 2022). However, no systematic review has been conducted on the implications of the COVID-19 pandemic on psychological and mental health aspects, considering the specificity of the sport and the characteristics of elite soccer players.

Psychosocial factors, including disturbances of attention and emotional balance, control of levels of arousal, anxiety, stress, daily annoyances, and other negative life events are predictive of the performance and mental health of soccer players. In this sense, it is imperative to verify whether interventions based on psychological approaches can reduce and mitigate the impacts of these factors on athletes during the pandemic. Based on empirical data, the analysis of the impact that the COVID-19 pandemic has on the psychological aspects and mental health of soccer athletes aims to promote a better understanding of this phenomenon and contribute to the development of guidelines based on evidence, so that clubs and soccer professionals can implement solutions and strategies to reduce the damages and preserve or even increase the performance of athletes in different dimensions in the sport. This will be particularly useful in helping to increase integrative approaches to athletes’ performance and health. Thus, the present systematic review aims to analyze the impact of the COVID-19 pandemic on the psychological aspects and mental health of elite soccer athletes.

## Methods

This is a systematic review of the literature that followed the criteria recommended by the PRISMA Declaration – Preferred Reporting Items for Systematic Reviews and Meta-Analyses (Page et al., 2021) registered in the International Prospective Register of Systematic Reviews–PROSPERO (CRD42022341545).

### Search strategy

The searches for articles were conducted in July 2023, in scientific journals indexed in the Cochrane, *PsycINFO, PubMed*, *Scopus*, *SPORTDiscus,* and *Web of Science* databases, with a search period from 1st January 2020 to July 11 2023, and the descriptors are presented in Table 1.

**Table 1:** Search strategy in electronic databases.

Terms	Descriptors
#1 Soccer	“Sport teams” OR Soccer OR Football OR footballer* OR “football play*” OR “soccer play*” OR “soccer athlet*”
#2 Covid/confinement	COVID-19 OR “Corona Virus Disease 2019” OR “Novel Corona Virus” OR “2019-nCoV” OR coronavirus* OR corona-virus* OR covid OR “social isolation” OR quarantine OR confinement OR “home-based” OR “disease outbreaks” OR outbreak OR lockdown OR “social distancing”
#3 Mental health, Psychology factors, Psychobiological Approach	mental* OR “mental health” OR psychol* OR depression OR anxiety OR burnout OR tension OR feeling* OR fear* OR panic* OR ptsd OR “post traumatic” OR distress OR “sport psycho*” OR rest OR fatig* OR recovery OR “under pressure” OR overtrain* OR overreach* OR detraininig OR burnout OR stress* OR distress* OR “subjective effort perception” OR SEP OR depress* OR anxiet* OR anxious* OR behavio* OR sleep* OR insomnia* OR mood* OR coping OR resilience OR flow OR emotion* OR “self-reported”
Combination	#1 AND #2 AND #3

The search and selection procedures of the articles were carried out by two researchers (WC and AD’O) independently. Any disagreements were resolved by a third party (AA).

### Eligibility criteria for studies

Only peer-reviewed studies were included, taking into account the following criteria: (i) the study was required to be related to the COVID-19 pandemic and present analyses of data from empirical investigations including soccer athletes in the context of training, competitive performance, injury treatment, as well as retired athletes or those who had left the sport; (ii) the article was required to be written in English, Spanish, or Portuguese, with full text available. Studies with only abstracts available were not included, due to the impossibility of performing a full analysis.

For this review, the eligibility criteria were based on the strategy – PECOS – Population, Exposure, Comparator, Outcome, and Study Design (Morgan et al., 2018; Table 2).

**Table 2:** Inclusion and exclusion criteria of the studies selected for the review.

Strategies		Inclusion criteria	Exclusion criteria
P	Population	Elite soccer athletes of all ages	Players recreational/leisure football, Atypical development; American football; Australian Rules; Rugby; Gaelic football and referees
E	Exposure	COVID-19 pandemic, lockdown, social isolation	Studies on outcomes with the COVID-19 pandemic
C	Comparison	Control group, pre and post, gender, age group, position of players, competitive level	_
O	Outcome	To analyze the psychological factors, approaches the psychobiological s in elite soccer athletes.	_
S	Study	Original, experimental, randomized or not, cross-sectional, longitudinal, qualitative peer-reviewed studies	Systematic reviews, theoretical studies, conferences.

### Study selection and data extraction

At this stage of the review, the *Rayyan* application, developed by the *Qatar Computing Research Institute* (Ouzzani et al., 2016), was used. The selection and extraction of data from the studies were carried out by two researchers (WC and AD’O) independently, and any disagreements were resolved by consensus discussion.

For the analysis and discussion of the results, the following data were extracted: characteristics of the sample (number of participants, sex, mean and age range, and competitive level of performance), instrument for evaluation of mental health variables and psychological aspects, type of study, research objective, year and language of publication, and main results.

**Figure 1:**
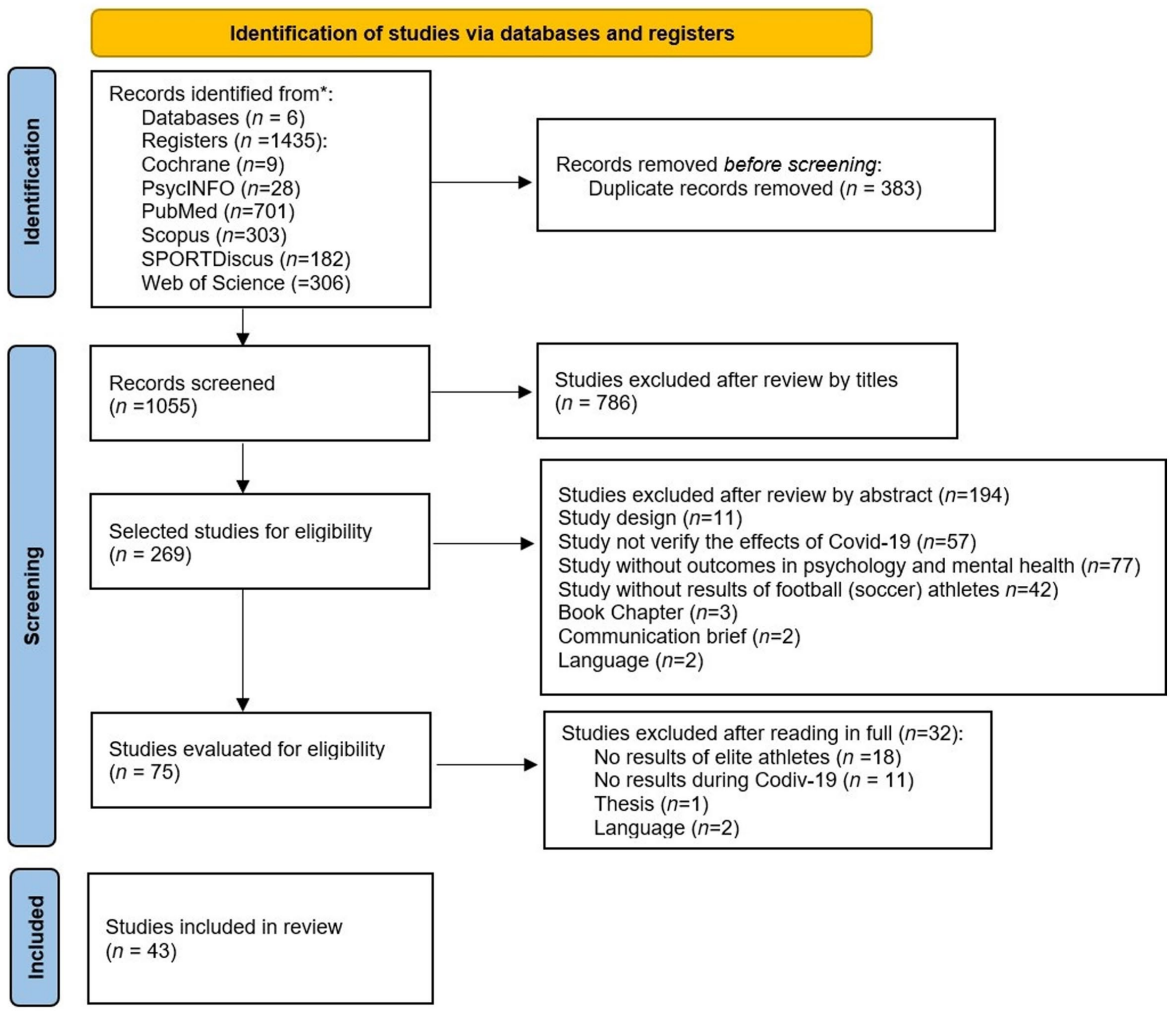
Study flowchart following PRISMA guidelines.

For the analysis of Figure 1, we considered the dates of completion of the studies included in this review and the data of cumulative cases and deaths from COVID-19 according to the World Health Organization (2020).

### Study quality assessment

The quality of the included studies was assessed using two tools – the Quality Assessment Tool for Observational Cohort and Cross-Sectional Studies and the Quality Assessment Tool for Before-After (Pre-Post) both from the National Institutes of Health (2021). Both tools include criteria that need to be met by the studies included in this review and at the end of the evaluation a score is assigned according to each criterion answered, for a “yes” answer, one point is added, and for “no,” “not applicable,” “not reported,” or “it is not possible to determine,” the value is assigned zero points. The Cohort and Cross-Sectional Studies tool – consists of an evaluation based on 14 criteria. A study-specific global score, ranging from zero to 14, was calculated by summing the scores of all criteria.

The tool for experimental studies (pre-post), contains 12 criteria, thus the overall score can vary from 0–12. A higher score indicates better quality of the study (National Institutes of Health, 2021). The evaluation was performed independently by two reviewers (WC and AD’O). Disagreements were resolved by consensus. When necessary, a third author (AA) was requested for the final opinion. The quality of the studies was not considered as an inclusion or exclusion criterion. Previous studies used these tools and demonstrated satisfactory applicability (Sarkies et al., 2017; Hayes et al., 2018).

The degree of agreement between the two evaluators was analyzed using the Cohen’s Kappa test. For the interpretation, the approach of Mchugh (2012) was used, which classifies the agreement as: None 0–4%; minimum 4–15%; weak 15–35%; moderate 35–63%; strong 64–81%; and nearly perfect 82–100% (McHugh, 2012).

## Results

The search strategy identified 1,435 studies. After the duplicates were removed, 1,055 studies remained. Screening of abstracts identified 75 potentially eligible studies. A total of 43 original studies met the inclusion criteria (Figure 1 PRISMA flowchart).

### Characteristics of the studies

From the 43 included studies, a general sample of 16,321 soccer athletes was identified in this systematic review, and the average number of participants was 388 athletes (7–16, 39). Most of the published studies include only soccer athletes in the sample (35/81.4%).

Researchers from diverse nations have contributed to scientific insights regarding the impact of the pandemic on the psychological aspects and mental health of soccer athletes. Among the 43 studies included in this review, the primary authors represent a wide array of countries: Turkey, Brazil, Chile, Italy, Spain, China, Iran, Germany, Australia, Austria, Belgium, Denmark, Egypt, the United States, France, the Netherlands, the United Kingdom, Norway, Slovenia, Serbia, and Sweden. This international collaboration underscores the global effort to comprehensively understand the effects of the pandemic on elite soccer athletes.

Some of the included studies (18/41.9%), did not report the country of origin of the athletes. Of the studies that reported the nationality, athletes of different nationalities were identified. Twenty studies (46.5%) included female and male athletes, 17 studies only male athletes, in three studies the athletes were only female (6.9%), and five studies did not report the sex of the participants. Most studies included adults and young people, representing 69.2% of the studies, except for two that included young athletes (mean age 12.6 years). Ten studies did not report the age of the participants.

Most studies included exclusively samples and data of soccer athletes (*n* = 34), and another nine studies included samples of soccer athletes with different sports modalities.

Regarding the study design, the majority were cross-sectional (42 of 43) and only one study design differs from the others, being non-randomized experimental. Seven studies presented a design with a longitudinal cross-sectional follow-up and three studies were cross-sectional and comparative (Table 3).

**Table 3:** Sample characteristics (number of athletes, sex, and age), study objectives and designs, athletes’ competitive level, result variables, instruments, and main results.

ID	Reference	Study design	Number of Athletes	Age – mean ± SD (min-max)	Sex (M/F)	Objective	Psychological and Mental Health Variables investigated	Athletes’ competitive level	Instrument	Main results
1	Esteves et al. (2020)	Cross-sectional	529	NR	M/F	To verify the associated factors of trait-anxiety and anxiety state in professional soccer teams during the COVID-19 pandemic.	Trait state of anxiety	NR/NR	State–Trait Anxiety Inventory (STAI); Self-Perceived Performance Scale (SPS)	The results highlight the importance of cognitive-behavioral therapy for professional soccer teams. Its core is cognitive restructuring using the ABC (antecedents-behavior-consequences) model
2	Evli et al. (2020)	Cross-sectional	620	NR	M	To examine the anxiety levels of soccer athletes in different leagues in relation to the variables of the training program.	Anxiety levels	NR/NR	Trait and State Anxiety Inventory	High-level athletes have a high state of anxiety due to uncertainty in a pandemic environment. It can be stated that this uncertainty increases the anxiety levels of these athletes, who practice the sport as a profession for reasons such as making money and keeping fit.
3	Håkansson et al. (2020)	Cross-sectional	1,145	15 years or older	M/F	To study the perceived psychological influence of the COVID-19 pandemic and symptoms of depression, anxiety, and changes in alcohol consumption, gambling behavior, and during lockdown.	Symptoms of depression, anxiety	NR/NR	Depressive symptoms were measured using the PHQ-9 (patient health questionnaire-9); anxiety symptoms were measured using GAD-7.	Depression and anxiety have been linked to feeling worse during the COVID-19 pandemic and worrying about one’s sporting future. Female athletes had a higher incidence of anxiety and depression symptoms. The fear of increased gambling during the crisis was not clearly demonstrated, but the risk in male athletes was common and associated with increased gambling during the pandemic.
4	Li et al. (2020)	Cross-sectional longitudinal	221	15.3 ± * (13–19)	M	To examine the changes in the coach-athlete relationship before and after lockdown	Interpersonal relationship coach/athlete	NR/NR	Coach-Athlete Relationship Questionnaire (CART-Q)	The mean values of the three dimensions of the coach-athlete relationship after lockdown are higher than before lockdown; blockage has different effects on proximity, commitment and complementarity at different ages; The relationship between the impact of lockdown on the coach-athlete relationship and the severity of the epidemic in each province is not significant.
5	Mon-López et al. (2020)	Cross-sectional	175	25.1 ± 4.89	M/F	To analyze the effects of lockdown during the COVID-19 pandemic on training and sleep and establish the relationship between emotional aspects and training based on sex and competitive level.	State of Mood and Emotional Intelligence	NR/NR	Profile of Mood States (POMS); Wong Law Emotional Intelligence Scale Short form (WLEIS-S)	COVID-19 reduced sleep quality, duration and intensity of training. Mood states can affect sleep quality, sleep hours, and perceived exertion assessed. Emotional Intelligence is related to the training of behaviors in periods of isolation. The period of isolation did not affect men and women equally.
6	Aghababa et al. (2021)	Cross-sectional	508	24.66 ± *	M/F	To investigate the impact of confinement on physical activity (PA) patterns before and during confinement among team sports participants.	Mood	Amateur and semi-professional athletes/NR	Brunel Mood Scale (BRUMS)	Given the complex psychosocial situation of COVID-19-related lockdown, it seemed very unlikely that unique patterns of physical activity could counterbalance possible impaired mood and behavior states.
7	Balyan et al. (2021)	Cross-sectional	306	20 years or younger and 31 years or older	M	To compare the relationship between nutrition and life satisfaction of soccer players in the COVID-19 period.	Life satisfaction	NR/NR	Life Satisfaction Scale; Three Factor Nutrition Scale (TFEQ)	Life satisfaction and nutrition were associated and are directly proportional. The nutritional habits of soccer players affect individuals. Soccer players who enjoy their life and feel psychologically happy produce effects on their nutrition and life satisfaction.
8	Esteves et al. (2021)	Cross-sectional	563	NR	M/F	To compare the factors that can increase trait and state of anxiety among professionals and soccer players during the COVID-19 pandemic according to the influence of sex.	Anxiety	NR/Between 1 to 20 years of experience	State–Trait Anxiety Inventory (STAI)	There are differences in anxiety state and anxiety trait, with higher results in men compared to women. Anxiety during the period of the COVID-19 pandemic impacted participants’ self-perceived performance analysis. The importance of cognitive-behavioral therapy for professional soccer teams is highlighted.
9	Facer-Childs et al. (2021)	Cross-sectional	565	(26-30)	M/F	To investigate how training and sleep-related factors have changed in a diverse group of athletes during the COVID-19 lockdown and whether these changes are associated with mental health symptoms.	Sleep and mental health	Elite and subelite athletes/NR	Ultra-Short Munich Chronotype Questionnaire (UMCTQ); Single Daytime Sleepiness Item; Patient Health Questionnaire-4 (PHQ-4); Perceived Stress Scale-4 (PSS-4); Morningness–Eveningness Questionnaire item 19 (MEQ19); Athlete Chronometric Evaluation (ACE)	Significant disruptions were reported for all lifestyle factors, including social interactions, physical activity, sleep patterns, and mental health among athletes. A significant increase in total sleep time and latency was found, as well as a delay in mid-sleep and a decrease in social jetlag. The frequency and duration of training decreased significantly.
10	Grimson et al. (2021)	Cross-sectional longitudinal	25	27,2 ± 4,0	M	To describe the fluctuations in mental well-being during lockdown and return to sport protocols compared to “normal” in professional soccer	Mental well-being	NR/NR	Warwick-Edinburgh Mental Wellbeing Scale (WEMWBS)	Mental well-being responses to the block were better understood individually. Physical activity only had a measurable effect on mental well-being when >250 min. The stressors imposed on players during a season are potentially greater than those inflicted by lockdown.
11	Han et al. (2021)	Cross-sectional	7	(30–32)	M/F	To address the severity and psychological support for athletes with COVID-19.	Mood, Stress, Anxiety, Sleep Disorder, Depression	Elite professional athletes/NR	Self-Reporting Questionnaire 20 (SRQ-20), Patient Health Questionnaire 9 (PHQ-9), Profile of Mood States (POMS), Generalized Anxiety Disorder 7 (GAD-7), Hamilton Depression Rating Scale (HAMD), and Hamilton Anxiety Rating Scale (HAM-A)	The severity and mortality in Chinese athletes contracted with COVID-19 are mild and low, with zero deaths. Psychological support of any kind from the nurses, medical staff, psychologists, family, and friends through social networks and communication should be adopted and can be of great help.
12	Ivarsson et al. (2021)	Cross-sectional longitudinal	101	22.4 ± 5.2	M/F	To describe the changes in mental health and well-being over an 8-week period of lockdown and restricted training during the COVID-19 pandemic in soccer players of different levels.	Well-being, positive and negative affects	Elite professional athletes/NR	WHO-5 Well-Being Index; The Positive and Negative Affects Schedule – Short version	A high number of players reported clinical levels of depressive symptoms compared to what was previously found in high-level athletes. The number decreased during the 8-week period. A similar trend was found for negative effects. Despite a higher prevalence of depressive symptoms at the onset of lockdown, this improved as players progressed to fewer restrictions.
13	Jansen (2021)	Cross-sectional	55	(16–46)	M/F	To investigate whether soccer athletes suffer from depressed mood and fear of the future.	Self-compassion, fear, anxiety, depression	NR/NR	Self-Compassion Scale (SCS); Rumination–Reflection Questionnaire (RRQ); Penn State Worry Questionnaire (PSWQ)	The results show an association between the negative scale of self-compassion and depressive mood, as well as fear of the future. While depressive mood was predicted by self-compassion, fear of the future was only indirectly predicted by self-compassion for the mediating effects of repetitive thinking. In addition, in semi-professional soccer, self-compassion interventions can be a useful tool in difficult times.
14	Jiménez-Barreto and Borges (2021)	Cross-sectional	19	17.63 ± 4.45 (13–34)	F	To evaluate the impact of PEVG (virtual group training program) on conditional stimulation, mood and psychosocial support.	Mood, well-being, psychosocial support	NR/NR	Instrument to verify the basic psychological needs; Questions related to well-being, produced by the authors.	Significant differences in load perception, showing higher scores for young women in relation to older women, and attackers in relation to defenders were identified in the scores at the end of the virtual group training program. These results advise that the normative parameters, specific position, and age in the follow-up of the training process be considered. A high score was found in the dimension support for social relations, which, taking into account the situation of separation of the players, is related to the use of the telematics application.
15	Kesilmis et al. (2021)	Cross-sectional	286	21.93 ± 4.72	NR	To examine the correlation between physical activity, mood, and nutrition of amateur soccer players and the differences in mood and nutritional variables depending on their physical activity status.	Mood	NR/Average of 9.41 years of experience	Brunel Mood Scale; International Physical Activity Scale; Three-Factor Eating Questionnaire	The results of this research showed that maintaining regular physical activity was an important preventive strategy for mental and nutritional health during the period of mandatory isolation in the current pandemic.
16	Leguizamo et al. (2021)	Cross-sectional	310	22,26 ± 4,98 (18–49)	M/F	To analyze the relationships between perfectionism and trait-anxiety with mental health indicators in high-performance athletes during lockdown and to explore the coping strategies that athletes applied and whether they are perceived as helpful in managing negative emotional states.	Mood, depression, state of anxiety and stress	Elite professional athletes/NR	Multidimensional Perfectionism Scale (FMPS); State–Trait Anxiety Inventory (STAI-T); Depression, Anxiety, and Stress Scales (DASS-21); Profile of Mood States; Approach to Coping in Sport Questionnaire (ACSQ-1); Sports Sleep Questionnaire (CSD in Spanish).	A strong relationship was observed between perfectionism and the sports discipline of martial arts, superior to other sports. The high-performance athletes in the sample studied presented negative emotional state values below the expected average.
17	Leitner and Richlan (2021)	Cross-sectional	NR	NR	NR	To analyze how the influence of “ghost games” (without fans) influences the experience and behavior of players, officials, and referees.	Emotional behaviors related to the game of football (discussion, fair play, name-calling)	NR/NR	Analysis System for Emotional Behavior in Football (ASEB-F)	The study provides unprecedented information about the effects of a lack of fans at soccer games during the COVID-19 pandemic on emotional behavior on the field. Without the external factor of the fans, players and staff acted more factually and got less excited. The evidence from this study indicates that the absence of fans has a substantial influence on the experience and behavior of players, staff, and referees.
18	Lima et al. (2021)	Cross-sectional	523	(18–32)	M/F	To assess the psychological state of professional men’s soccer players who have been infected by COVID-19.	Anxiety, fear, depression, stress, psychological suffering	NR/NR	Depression anxiety stress scale-21 (DASS-21); Kessler Psychological Distress Scale (K-10)	COVID-19 infection negatively affects the psychological states of soccer players. Among athletes who have been infected by COVID-19, it is recommended that these should be closely monitored, with psychological support.
19	Madsen et al. (2021)	Cross-sectional longitudinal	125	NR	M	To investigate the protective role of resilience and the level of anxiety of elite soccer players.	Sports resilience and anxiety	NR/NR	The Sport Anxiety Scale-2 (SAS-2); The Connor-Davidson Resilience Scale (CD-RISC-25); The World Health Organization-5 well-being index (WHO-5); The short version of The Positive and Negative Affect Schedule.	The results show a positive relationship between the average level of well-being and positive affect and intrapersonal variability over time. This indicates that resilience can be a protector for mental health. In addition, higher levels of anxiety trait were associated with higher levels of negative affect and greater variability over time.
20	Mehrsafar et al. (2021)	Cross-sectional	90	26.33 ± 2.48	M	To examine the relationship between competitive anxiety, fear of COVID-19, and autonomic and endocrine stress responses in professional soccer players after returning to competition during the COVID-19 pandemic.	Fear, anxiety and stress	NR/Average 10.03 years of experience	Competitive State Anxiety Inventory-2 Revised (CSAI-2R); Fear of COVID-19 Scale (FCS); Coronavirus Anxiety Scale (CAS)	Preliminary evidence indicates that anxiety caused by COVID-19 and competitive anxiety can have a negative impact on the athletic performance of professional soccer players during COVID-19 pandemic competitions.
21	Nassar et al. (2021)	Cross-sectional	37	10.8 ± 0.46 (9–11)	M	To highlight the physical and psychological health risks of an Egyptian soccer team faced during the first lockdown of the COVID-19 wave.	Depression, anxiety, stress	NR/NR	The Children’s Sleep Habits Questionnaire (CSHQ); Depression Anxiety Stress Scale (DASS-21)	The mean quality of life (QoL) score of the athletes worsened significantly and a significant negative correlation was found between the increase in body mass index (BMI) and QoL. There was a significant increase in BMI among athletes who did not exercise at home and a negative correlation with the change in QoL over the course of the blockade. The mothers’ anxiety had a possible reflection on their children’s weight gain.
22	Şenışık et al. (2021)	Cross-sectional	571	24.53 ± 5.09	M/F	To explore whether the mental health states of professional athletes were affected by the isolation period when sports were suspended due to the pandemic.	Depression, anxiety and stress	NR/NR	Depression Anxiety Stress Scale 21 (DASS-21), The Impact of Events Scale-Revised (IES-R) and International Physical Activity Questionnaires (IPAQ)	The mental health status of the athletes was better than the non-athletes, and the positive effect of the sport, which was made until the break due to the isolation period, continued on mental health. These findings show that physical activity and sports can help protect mental health.
23	Smith (2021)	Cross-sectional longitudinal	376	NO	M	To explore how soccer players as athletes helped during the pandemic and their role and support to the community.	Citizenship, community support, well-being	NR/NR	NA	The findings show 12 athletes with citizenship roles during the pandemic, which collectively illustrate players promoting fan support and citizen public health compliance, well-being and life. The players also conveyed how they handled the pandemic with their athlete mindset and hopes for a brighter future.
24	de Souza et al. (2021)	Cross-sectional	24	18.4 ± 1.4	M	To analyze the impacts of COVID-19 preventive measures on symptoms of anxiety, stress and depression in professional soccer athletes and see if experience time interferes with the outcomes.	Anxiety, stress and depression	Elite professional athletes/NR	Depression and Stress-21 Scale (DASS-21), Competitive State Anxiety Questionnaire (CSAI-2 R)	
25	Sun et al. (2021)	Cross-sectional longitudinal	139	21.33 ± 4.75	M/F	To examine whether the daily visionary leadership behavior by their supervisors alleviates the adverse impacts of the daily intrusive experience of COVID-19.	Depression and anxiety	NR/NR	Patient Health Questionnaire (PHQ-4); Psychosocial resource loss and gain were measured daily.	During the pandemic, there was no variation in the levels of symptoms of anxiety, stress, and depression in soccer athletes. The return to training, after the period of interruption of training, reduced the levels of somatic anxiety, cognitive anxiety, depression, stress, anxiety and self-confidence, but without significant differences. It was identified that athletes with more than 10 years of practice had a significant increase in confidence compared to athletes with less than 10 years of practice.
26	Mavi Var et al. (2021)	Cross-sectional	1,176	NR	NR	To analyze some issues faced by active athletes during the period of isolation of COVID-19.	Well-being	NR/NR	Open questions developed by the authors were used as a data collection instrument.	The measures collected daily presented considerable variations over the days, the proportion of variation within the person was 69% for intrusive COVID-19 experience, 63% for visionary leadership behavior, 78% for loss of psychosocial resources, 79% for gain in psychosocial resources, 77% for mental health status according to depression and anxiety scores, and 71% for performance by depression and anxiety scores, and 71% for work performance. The daily experience of the pandemic affects daily mental health and job performance status. Visionary leadership could help alleviate the adverse impact of COVID-19
27	Villaseca-Vicuña et al. (2021)	Cross-sectional	32	26 ± 4	F	To determine the impact of COVID-19 lockdown measures on the training load and well-being of professional soccer players in Chile.	Well-being, stress level and mood	NR/Average 7.0 years of experience	The Perceived Exertion Scale (EPE) evaluates: well-being, perceived fatigue, muscle pain, stress level and mood.	The isolation of COVID-19 led to limitation of movement of the athletes and caused them to face a series of physical, physiological, and psychological problems because they were forced to continue training at home and with their own equipment, which ultimately resulted in reduced performance. 7% of athletes stated that their performance decreased during the COVID-19 isolation period while 25.3% of them reported no drop in performance.
28	Wagemans et al. (2021)	Cross-sectional	33	NO	M	To examine the influence of COVID-19 on physical performance and mental health in professional soccer athletes during the 2020–2021 season.	Well-being, stress level and mood	NR/NR	Hooper Scale (the club’s medical staff of modified this scale by adding an extra variable “mood-score” and changing the score range from 0 to 6)	The participants’ level of well-being was negatively affected by the lockdown. Although the perception of the training load was not affected, it is not possible to state that a longer period of confinement would not reduce it, resulting in a decrease in performance.
29	Zilli et al. (2021)	Cross-sectional comparative	50	19.78 ± 1.22 (18–23)	M/F	To investigate the prevalence rates of anxiety and depression in a sample of college athletes; (2) to examine the relationships between the use of psychological strategies (PE) by athletes in training and competitions with risks of anxiety and depression; and (3) explore associations between the variability of athletes’ history in the use of PE and mental health risks.	Anxiety and depression	NR/Average 13.0 years of experience	Beck Anxiety Inventory, Center for Epidemiologic Studies Depression Scale, Test of Performance Strategies (TOPS)	Physical performance varied considerably between results before and 8 weeks after COVID-19 infection in a sample of Division I soccer players. However, the soccer players’ overall well-being, stress levels, and mood decreased after a positive test for COVID-19.
30	Dönmez et al. (2022)	Cross-sectional	977	27.2 ± 5.3	M	To assess the psychological impacts of lockdown and similar restrictions on professional soccer players during the coronavirus pandemic.	Depression	Elite professional athletes/NR	Center for Epidemiologic Studies Depression Scale (CES-D), Impact of Event Scale-Revised Scores (IES-R) and short form of International Physical Activity Questionnaire (IPAQ)	The results indicated that college athletes at no risk for anxiety or depression used more Psychological Strategies (PE), while those at risk reported using debilitating strategies. Lack of emotional control in practice explained the greatest variation in predicting anxiety scores (29%) and depression (36%), while negative thinking in competition explained the greatest variation in anxiety (30%) and depression (35%) scores. The results are discussed in terms of athletes’ familiarization with the variety of PE and its relevance to mitigating mental health risk triggers during the pandemic.
31	Gouttebarge et al. (2022)	Cross-sectional comparative	1,602	NR	M/F	To establish the prevalence of anxiety and depressive symptoms among professional soccer players (i.e., soccer; hereinafter “soccer”) during the COVID-19 emergency period by making comparisons with players evaluated prior to exposure to the COVID-19 emergency period.	Anxiety and depression	Elite professional athletes/NR	Generalized Anxiety Disorder (GAD-7); Depressive symptoms were measured with the Patient Health Questionnaire (PHQ-9)	While the findings look promising about higher levels of physical activity in professional soccer players during home quarantine, stays at home can contribute to anxiety and depression. Maintaining regular physical activity and routine physical exercise in a safe home environment is one of the most important strategies for maintaining a healthy mental state.
32	Katanic et al. (2022)	Cross-sectional	360	21.71 ± 4.42	M	To determine the structure of motivation for sports practice in soccer players during the period of the COVID-19 pandemic.	Motivation	Elite professional athletes/5 years or more of experience	Questionnaire on the motivation for participation in sport modified according to PMQ.	The COVID-19 emergency period is associated with increased symptoms of anxiety and depression in professional soccer players, especially among those worried about their future as players.
33	Keemss et al. (2022)	Non-randomized trial	8	(16–19)	M	To investigate the effects of a 13-week supervised home training program on physical performance, sleep quality, and health-related quality of life in young professional soccer players during the first COVID-19 lockdown in Germany.	Sleep quality and Quality of life	Professional Youth Athletes/NR	Short-Form36 Health Survey (SF-36); Pittsburgh Sleep Quality Index (PSQI)	‘Sports success’, ‘Support’, ‘Social status’, ‘Friendship’, ‘Physical health’, and ‘Sports activities’. These six dimensions explain the space of participation of the total manifest motivation with 62.26% of the total variance. These results contributed to understanding of the motivational structure of professional soccer players for sports practice. The main motivational dimensions for engagement and commitment to sport during the COVID-19 pandemic were pointed out.
34	Kobal et al. (2022)	Cross-sectional	24	28.0 ± 4.6	F	To compare the effect of a new rule for substitutions (four and five) with the pre-COVID-19 pandemic rule (up to three) on recovery status, physical and technical performance, internal workload, and recovery process in elite women’s soccer players.	Recovery	NR/NR	Total Quality Recovery (TQR); Rating of Perceived Exertion (RPE); Match Running Performance	The monitored home training program was effective in maintaining muscle strength, endurance, performance, and sleep quality during the COVID-19 lockdown. The player’s body weight, body mass index, and body fat increased significantly, as did the mental health component of the SF-36. Physical performance and sleep quality could be maintained during the home training period. Training planning when players return to their normal training after 13 weeks of training at home can help coaches create training programs for young professional soccer players on longer breaks from soccer practice interruption or in case of another lockdown.
35	Lima et al. (2021)	Cross-sectional	261	(18–32)	M/F	To evaluate the association between mental health problems, demographics, and SARS-CoV-2-related variables in soccer players infected with SARS-CoV-2.	Depression, anxiety and psychological suffering	NR/NR	Athlete Psychological Strain Questionnaire; Depression Anxiety Stress Scales-21	No differences were observed in any physical and technical parameters between 4–5 and ≤ 3 substitutions (*p* > 0.05). In addition, 4–5 substitutions showed lower RPE (*p* < 0.001) and load RPE (p < 0.001), higher TQR (*p* = 0.008), and shorter time played by the player (p < 0.001), compared to ≤3. Thus, the new interim rule for replacements improved the balance between stress and recovery during the COVID-19 pandemic.
36	Marholz et al. (2022)	Cross-sectional	94	24 ± 5.6	NR	To explore trait-anxiety levels, feelings of well-being, and the relationship between both variables in professional soccer players from four clubs in Chile	Anxiety and well-being	NR/NR	State-Tear Anxiety Inventory (STAI); PERMA-Profiler measures the five pillars of well-being.	Mental health (SM) problems in infected soccer players were associated with female players, participation in soccer at the lower level, performance concerns, and sleep problems. The evaluation of MS is indicated in infected athletes to assist in the detection and appropriate intervention.
37	Merino-Muñoz et al. (2021)	Cross-sectional	28	26 ± 6.3	M	To determine the effects of COVID-19 lockdown measures on the degree of well-being of professional soccer players.	Well-being	NR/NR	Hooper Scale “Well-being questionnaire”	the soccer players investigated have average levels of trait-anxiety and a good level of general well-being, with a correlation between the two. Although the current situation can be seen as threatening, the maintenance of working and active conditions for an athlete seems to be fundamental to feel a certain degree of control in the face of uncertainty, as well as coping strategies that allow a high sense of well-being.
38	Paravlic et al. (2022)	Cross-sectional longitudinal	266	22.59 ± 3.91	M	To examine the effects of the confinement period on basic anthropometry measurements, jump-against-motion performance, contractile properties of skeletal muscle derived from tensiomyography, incidence of injuries, and self-assessment of the general well-being of elite soccer players.	Well-being	NR/NR	The Lockdown Impact Characteristics Questionnaire Survey	The state of well-being in professional soccer players is altered in confinement in relation to competition, with significant changes in perceived fatigue, sleep quality, and level of stress and general well-being, which coincided with moderate to very large variation in these same variables, as well as the a percentage of change that was greater than the coefficient of variation between periods which could be an effect attributable to mandatory isolation.
39	Pété et al. (2022)	Cross-sectional	526	21.87 ± 8.66	M/F	To identify athlete coping profiles using a person-centered approach, based on the reported use of various coping strategies in response to the impact of the COVID-19 outbreak and compare anxiety levels, stress assessments, interpersonal coping strategies, and availability and appreciation of key sources of support across all profiles.	Coping profiles (self-sufficient, engaged, avoidant, active and social), anxiety, stress and social support interpersonal coping	NR/NR	Stress Appraisal Measure; The 28-item Brief COPE; Adapted version of the social support scale for the professional domain; Communal Coping Strategies Inven tory for Competitive Team Sports	Jump performance did not change between pre- and post-lockdown. The athletes rated the lockdown period as a positive event and felt better psychologically during the lockdown as they spent more time with family and friends. Although there were no differences in any of the variables that describe the muscle power of the lower limbs after the two-month blockade, the altered contractile properties of the muscles evaluated suggest suboptimal conditioning of the soccer players.
40	Vitali et al. (2022)	Cross-sectional	672	(18-45)	M/F	To gain an understanding of the impact of the COVID-19 pandemic on the well-being and sports readiness (training and competition) of female competitors and male athletes practicing outdoor and indoor team sports who were active during the first and second waves of COVID-19 in Italy.	Fear	NR/3 years or more of experience	World Health Organization Well-Being Index; Sport Readiness	The four coping profiles differentiate distinct groups of athletes in relation to anxiety, stress assessments, social support, and interpersonal coping. Evasive profiles were characterized less. Managing the COVID-19 situation may be more problematic for them than others in mitigating its negative psychological effects. Using a person-centered approach, the findings may inform the development of more appropriate care, support, and intervention for athletes, especially evasive ones, who have been characterized by less effective coping skills and resources.
41	Zago et al. (2022)	Cross-sectional comparative	1,163	(12–17)	M/F	To describe the physical activity and quality of life during the April 2020 lockdown of young participants in organized soccer.	Perceived quality of life	NR/NR	Youth Quality of Life Instrument – Short Form (YQoL-SF); Physical Activity Questionnaire for Adolescents (PAQ-A)	Indoor team sports and female athletes showed higher perceived risk of COVID-19, while athletes with no experience with COVID-19 reported greater fear. Perceived risk (directly and via perceived safety of the training environment) and fear of COVID-19 were negatively associated with athletes’ well-being and athletic readiness.
42	Montealegre-Mesa et al. (2023)	Cross-sectional	90	24.0 ± 4.2	M	To determine the training load and its relationship with the psychological response of professional soccer players during the lockdown due to the COVID-19 pandemic.	Mood State	NR/13 years or more of experience	POMS	There was a reduction in physical activity levels, with 56% of participants reporting seven or more hours/week of moderate to vigorous physical activity, which decreased with age and changed according to geographic context. Perceived quality of life was lower in young people playing in non-elite clubs and in older girls; Coaches, more than official initiatives, were the main source of soccer exercises practiced at home.
43	Romdhani et al. (2023)	Cross-sectional	1,639	24.0 ± 5.7	M/F	To explore the effects of soccer from the COVID-19 lockdown in early 2020 on the sleep and training loads of an international sample of soccer players	Sleep	Elite and non-elite professional athletes/NR	Pittsburgh Sleep Quality Index PSQI, (2) Insomnia Severity Index (ISI)	The soccer players trained on average 5.4 ± 1.28 days a week and practiced 2.4 ± 0.64 h a day and in isolation 1.68 ± 0.79 h with training frequency of 1.4 times a day. Changes in mood states were inversely correlated with demand and training. There was a significant correlation between the time of previous daily training and in isolation and also with the weekly schedule during preventive isolation. Important changes in the mood states of soccer athletes were observed during the lockdown caused by the COVID-19 pandemic.

Does not report age standard deviation: *; NR, Does not report; NA, Not applied; M, Male; F, Female.

### Characteristics of the instruments used

Different instruments were used to assess soccer athletes during the pandemic. Of the 43 studies included in this review, only one study did not describe the instrument used (Smith, 2021) Among the instruments applied in the published studies 39 were identified and widely used in studies with athletes, and three instruments were developed exclusively for research during the pandemic.

Studies on elite soccer athletes that examine the psychological impacts of the COVID-19 pandemic have extensively utilized well-established assessment tools, reflecting a rigorous approach to understanding the nuances of mental health. Notably, instruments such as the State–Trait Anxiety Inventory (STAI) and Profile of Mood States (POMS) have been employed to gauge anxiety states, depression, and mood variations among athletes. Additionally, widely recognized scales, such as the Patient Health Questionnaire (PHQ-9), Generalized Anxiety Disorder 7 (GAD-7), and Beck Anxiety Inventory have been adopted to specifically assess depressive and anxious symptoms.

Researchers have also recognized the unique psychological challenges within the sports context, leading to the utilization of specialized assessments. The Competitive State Anxiety Inventory-2 Revised (CSAI-2R) and the Instrument to verify the basic psychological needs exemplify this tailored approach, aiming to capture the distinctive psychological nuances experienced by elite soccer athletes.

In pursuit of a holistic understanding of the pandemic’s psychological impacts, some studies have taken a pioneering stance by developing customized instruments and employing novel methodologies. These include the creation of open-ended questions by the authors and the adaptation of questionnaires to meet the specific demands of the study. This adaptive strategy underscores the necessity for tools tailored to the unique circumstances of elite soccer athletes, offering a more nuanced comprehension of the psychological repercussions of the COVID-19 pandemic.

### Athlete monitoring period during the COVID-19 pandemic

**Figure 2:**
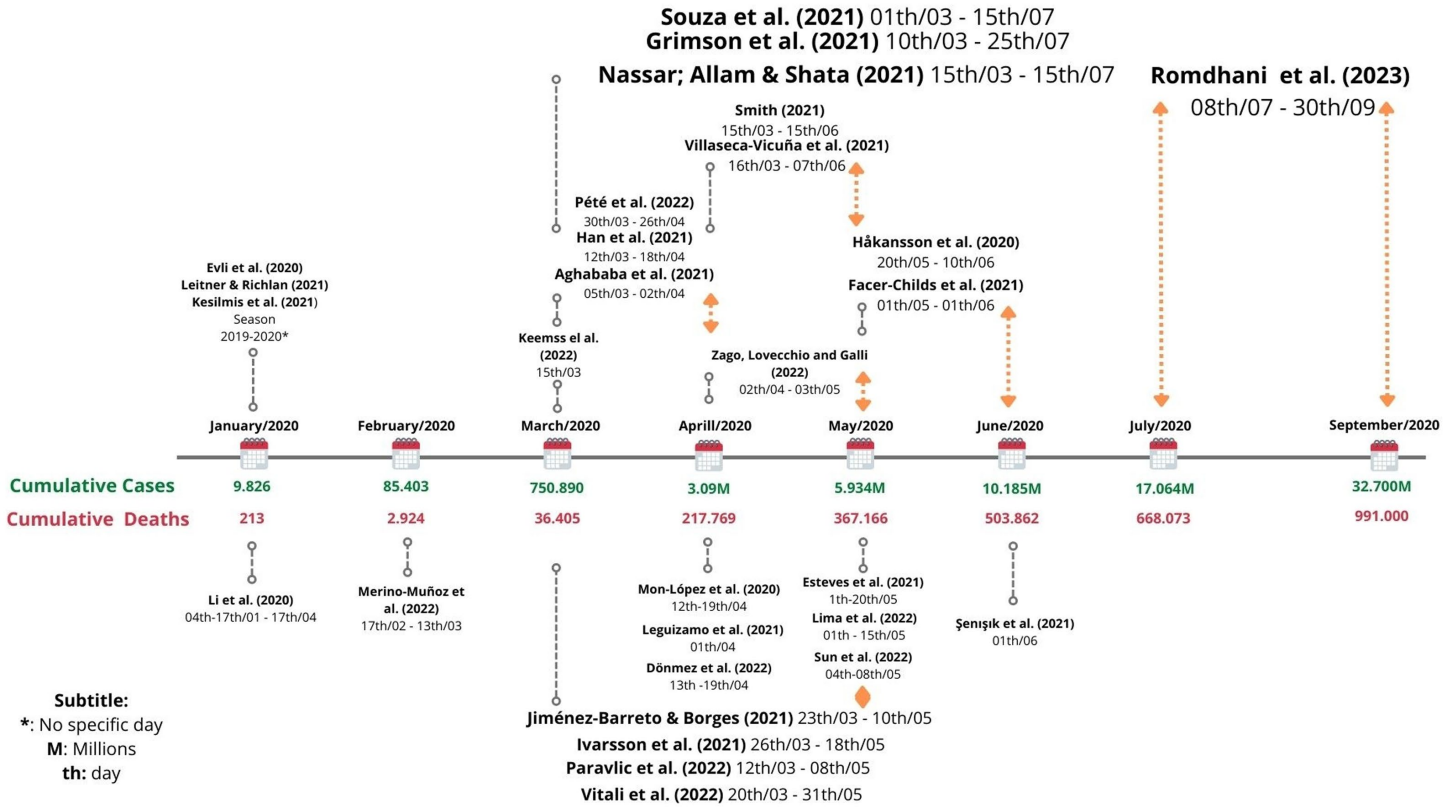
Timeline of the periods when the studies were developed and the monitoring of soccer athletes during the COVID-19 pandemic. Adapted from Richardson et al. (2022). Cumulative case and death data are derived from the closest update provided by the World Health Organization (WHO, 2020) on the last day of each month.

Most studies (29 out of 43) indicated the exact period of the pandemic in which the research was conducted. The majority of studies focused research on soccer athletes between March and June 2020 (23 out of 29), which is a period with a known high worldwide cumulative death rate of COVID-19, 540.267 cases. Five studies were conducted before this period (Figure 2).

### Anxiety

The most widely investigated psychological variable was anxiety, representing 20.45% of the studies. Anxiety levels were the most common measure in the studies (Esteves et al., 2020, 2021; Evli et al., 2020; Håkansson et al., 2020; Ivarsson et al., 2021; Lima et al., 2021; Madsen et al., 2021; Mehrsafar et al., 2021; Wagemans et al., 2021). The results of most of the studies demonstrated increased anxiety during the COVID-19 pandemic.

### Mental well-being

Mental well-being responses during the pandemic were investigated in 15.91% of studies (Grimson et al., 2021; Ivarsson et al., 2021; Jiménez-Barreto and Borges, 2021; Mavi Var et al., 2021; Merino-Muñoz et al., 2021; Smith, 2021; Villaseca-Vicuña et al., 2021; Wagemans et al., 2021; Marholz et al., 2022; Paravlic et al., 2022) The implications of the pandemic on the monitoring of mental well-being in soccer players present contradictory results and are discussed below.

### Mood state

Eight studies (Mon-López et al., 2020; Aghababa et al., 2021; Han et al., 2021; Kesilmis et al., 2021; Villaseca-Vicuña et al., 2021; Wagemans et al., 2021; Montealegre-Mesa et al., 2023) assessed the mood swings of soccer athletes during the COVID-19 pandemic. Aghababa et al. (2021) found that due to the psychosocial situation of the COVID-19-related lockdown, the maintenance and execution of unique patterns of physical activity were not compensatory to the point of producing positive effects and changes in the mood states of soccer athletes (Aghababa et al., 2021).

### Depression

Studies that analyzed depression in soccer players (13.64%), reported that during the emergency peak of the lockdown caused by the COVID-19 pandemic there was a significant increase in depression in elite soccer players, especially among those concerned about their future as players (Han et al., 2021; Leguizamo et al., 2021; Lima et al., 2021; Nassar et al., 2021; Dönmez et al., 2022).

### Sleep quality

The evaluation of the sleep quality of the athletes was investigated in four studies and significant changes were observed in the duration and quality of sleep of soccer athletes (Facer-Childs et al., 2021; Han et al., 2021). The effects of home training during lockdown brought positive results in the sleep of athletes (Keemss et al., 2022; Romdhani et al., 2023).

### Other psychological outcomes

**Figure 3:**
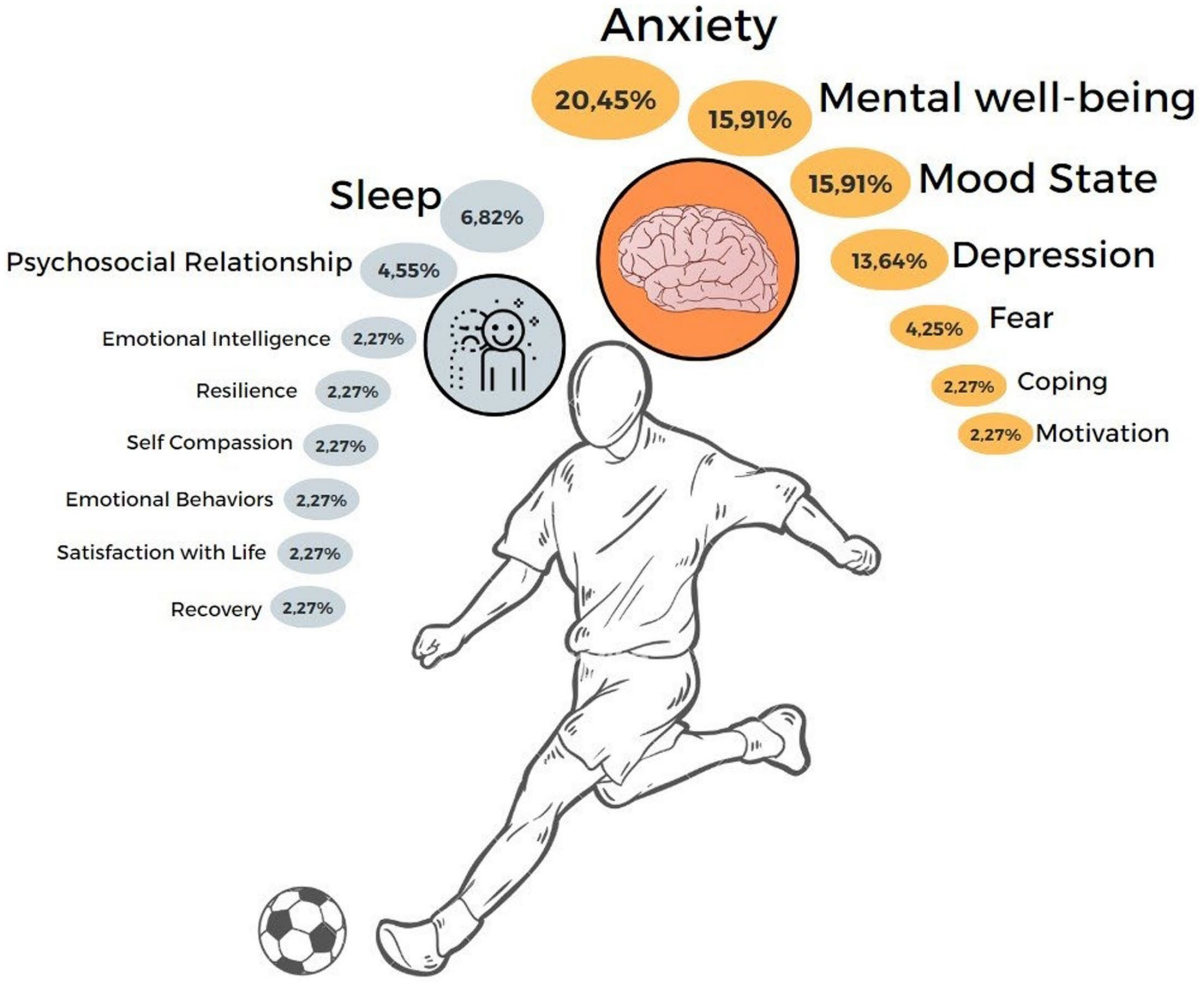
Frequencies of psychological and mental health variables investigated in the included studies (*n* = 43).

Studies were identified that investigated soccer athletes and other less frequent outcomes, such as coping, resilience, psychological distress, and fear caused by the pandemic (Jansen, 2021; Lima et al., 2021; Katanic et al., 2022; Kobal et al., 2022; Vitali et al., 2022). All health outcomes identified in the studies included in this review and their frequencies are shown in Figure 3.

### Evaluation of the quality of studies

Tables 4 5 present the general and specific classifications of the criteria on the evaluation of the quality of the studies (National Institutes of Health, 2021). The included observational studies scored an average of 7.00 (±2.26), ranging from 3 to 12. Based on this evaluation, the quality of the studies was generally considered regular (33 of 42), with 5 considered as of good methodological quality and four studies considered poor in the evaluation of quality. The agreement between the observers for all items was 85.6% (Table 4).

**Table 4:** Evaluation of the quality of cross-sectional and longitudinal studies.

ID studies	Criterion														Total score	Quality rating
	1	2	3	4	5	6	7	8	9	10	11	12	13	14		
1	1	1	1	1	1	0	0	0	1	0	1	0	0	0	7	Regular
2	1	1	1	1	0	0	0	0	1	0	1	0	0	0	6	Regular
3	1	1	1	1	1	0	1	0	1	0	1	0	0	0	8	Regular
4	1	1	1	1	1	1	1	0	1	1	1	1	1	0	12	Good
5	1	1	1	1	1	0	0	0	1	1	1	1	0	0	9	Regular
6	1	1	1	1	0	1	1	0	1	1	1	1	1	0	11	Good
7	1	1	1	1	1	0	0	0	1	0	1	0	0	0	7	Regular
8	1	1	1	1	1	0	0	0	1	0	1	0	0	0	7	Regular
9	1	1	1	1	0	0	0	0	1	0	1	0	0	0	6	Regular
10	1	1	1	1	0	1	1	0	1	1	1	0	0	0	9	Regular
11	1	1	1	1	0	0	0	0	1	0	1	0	0	0	6	Regular
12	1	1	1	1	0	1	1	0	1	1	1	1	1	0	11	Good
13	1	1	1	1	0	0	0	0	0	0	1	0	0	0	5	Regular
14	1	1	1	1	1	1	1	0	1	1	1	1	0	0	11	Good
15	1	1	1	1	1	0	0	0	1	0	1	0	0	0	7	Regular
16	1	1	1	1	1	0	0	0	1	0	1	0	0	0	7	Regular
17	1	0	0	0	0	1	1	1	1	1	0	0	0	0	6	Regular
18	1	1	0	1	1	0	1	0	1	0	1	0	0	0	7	Regular
19	1	1	1	1	0	1	1	0	1	1	1	0	0	0	9	Regular
20	1	1	1	1	0	0	0	0	1	0	1	0	0	0	6	Regular
21	1	1	1	1	0	0	0	0	1	1	1	1	0	0	8	Regular
22	1	0	0	0	0	0	0	0	1	0	1	0	0	0	3	Poor
23	1	1	0	1	0	0	0	0	0	0	0	0	0	0	3	Poor
24	1	1	1	1	1	0	0	0	1	0	1	0	0	0	7	Regular
25	1	1	0	1	1	1	0	1	1	1	1	0	0	0	9	Regular
26	1	0	0	0	0	0	0	0	1	0	1	0	0	0	3	Poor
27	1	1	1	1	1	0	0	0	1	1	1	0	0	0	8	Regular
28	1	1	1	1	0	0	0	0	1	1	1	0	0	0	7	Regular
29	1	1	0	1	0	0	0	0	1	0	1	0	0	0	5	Regular
30	1	1	0	1	0	0	0	0	1	0	1	0	0	0	5	Regular
32	1	1	1	1	1	0	0	0	1	0	1	0	0	0	7	Regular
33	1	1	1	1	0	0	0	0	0	0	1	0	1	0	6	Regular
34	1	1	0	1	0	0	1	1	1	1	1	0	0	0	8	Regular
35	1	1	0	1	0	0	0	0	1	0	1	0	0	0	5	Regular
36	1	1	1	1	0	0	0	0	1	0	1	0	0	0	6	Regular
37	1	1	1	1	0	0	0	0	1	0	1	0	0	0	6	Regular
38	1	1	1	1	0	0	0	0	1	1	1	0	1	0	8	Regular
39	1	0	0	0	0	0	0	0	1	0	1	0	0	0	3	Poor
40	1	1	0	1	1	0	0	0	1	0	1	0	0	0	6	Regular
41	1	1	0	1	0	0	0	0	1	0	1	0	0	1	6	Regular
42	1	1	0	1	0	0	0	1	1	0	1	0	0	0	6	Regular
43	1	1	1	1	1	1	1	1	1	1	1	0	0	1	12	Good
Agreement % (Kappa)	100	100	97,6	100	92,8	95,2	95,2	100	100	100	86.7	92,8	100	100	85,6	
Kappa interpretation (agreement)	Almost perfect	Almost perfect	Almost perfect	Almost perfect	Almost perfect	Almost perfect	Almost perfect	Almost perfect	Almost perfect	Strong	Strong	Almost perfect	Almost perfect	Almost perfect	Strong	

It cannot or cannot determine or cannot be determined or unreported (0); Yes (1); The quality of the studies was classified as poor (0–4 in 14 questions), regular (5–10 in 14 questions) or good (11–14 in 14 questions).

**Table 5:** Evaluation of the quality of the experimental study.

ID studies	Criterion												Total score	Quality rating
	1	2	3	4	5	6	7	8	9	10	11	12		
33	1	1	1	1	1	1	1	0	1	1	0	0	9	Regular
Agreement % (Kappa)	100	75	50	75	50	75	100	100	25	100	100	100	86.5	
Kappa interpretation (agreement)	Almost perfect	Strong	Moderate	Strong	Moderate	Strong	Almost perfect	Almost perfect	Almost perfect	Almost perfect	Almost perfect	Almost perfect	Strong	

It cannot or cannot determine or cannot be determined or unreported (0); Yes (1); The quality of the studies was classified as poor (0–4 in 12 questions), regular (5–10 in 12 questions) or good (11–12 in 12 questions).

The majority of the observational studies (37 out of 42) did not report the exposure levels of study participants in relation to the results, some (26 out of 42) also reported no information on sample power or size because the analyses are exploratory in nature. It was also observed that only two studies of the 42 evaluated indicated the appropriate statistical procedures to minimize confounding factors. Well-conducted cohort studies control for multiple potential confounding factors.

The experimental study included in the review presented a score of nine, considered regular quality. In the current study, statistical tests were presented to provide *p*-values for pre-post changes. The study provided sufficient information about the intervention in a consistent manner regarding the study population, as well as making all the measures performed on the study participants available in a clear and reliable manner. On the other hand, the calculation of the sample size was not provided, nor were the people who evaluated the results blinded to the exposures/interventions of the participants in any of the studies. Outcome measures were evaluated before and after the intervention. The agreement between observers for all items was 86.5% (Table 5).

## Discussion

### Overview and highlights

The present study aimed to analyze the impact of the COVID-19 pandemic on the psychological aspects and mental health of elite soccer athletes. Although a systematic review has recently been conducted including several sports modalities (Jurecka et al., 2021) to evaluate the effects of the pandemic on the physical activity, mental state, and quality of life of professional athletes, there is a need to deepen the searches and analyses, especially considering the most widely practiced and investigated modality in sports science (Kirkendall, 2020).

Regarding the nationality of the athletes investigated, 21 studies reported the origin of the athletes, who came from several different countries of the world. These data reveal the concern for the psychological aspects and mental health of soccer athletes in much of the world during the pandemic and related restrictions (de Souza et al., 2021; Kesilmis et al., 2021; Leguizamo et al., 2021; Keemss et al., 2022).

The current systematic review reports recent findings and summarizes the impact of the COVID-19 pandemic on soccer athletes, presenting rates of the main variables of psychological aspects and mental health measured during the pandemic and also showing a comprehensive approach in the selection of assessment tools for evaluating soccer athletes, emphasizing the importance of collecting not only overt symptoms but also sport-specific and individual factors experienced during the pandemic (Bernabe-Valero et al., 2021). The diverse array of instruments utilized contributes to richer understanding of the psychological impact of COVID-19 on elite soccer athletes, providing valuable insights for future research and interventions in sports science (Lee et al., 2023).

This multifaceted approach not only enhances our understanding of the subject within the specific context of elite soccer but also holds significance for its potential applicability across diverse populations and nationalities (Richardson et al., 2022). The inclusion of various instruments in the study not only enriches the depth of the analysis, but also increases the possibility of applying the findings in future studies.

Studies with heterogeneous samples have been reported in previous reviews focusing on the general population. Even so, considering the population of soccer athletes, it is important to reduce the risks of selection bias by considering homogeneous samples (Crossley et al., 2020).

### Impact of COVID-19 on psychological aspects in soccer athletes

Undoubtedly, the stress generated by the need to maintain physical condition prior to the pandemic caused imbalances in the stimulus-recovery relationship (Faude et al., 2014). Perceived stress is a critical factor in burnout among athletes and a moderate effect size was reported for the correlation of this variable with depression (Sarmento et al., 2021). A systematic review and meta-analysis prior to the pandemic synthesized, in an analytical and rigorous manner, data on psychological and hormonal changes induced by soccer matches. When psychophysiological stress was assessed after soccer matches, cortisol changes were caused by alterations in cognitive anxiety, while testosterone changes were driven by alterations in mood state and social connection. In addition, it is highlighted that competitive levels should be considered as the main moderating variables of the relationship between soccer games and hormonal changes related to stress (Slimani et al., 2017).

Understanding factors such as athletes’ sleep quality, and other psychological variables, such as resilience and coping strategies is essential to develop effective management protocols that help soccer athletes maintain healthy sleep and, consequently, optimize their athletic performance (Bicalho et al., 2020; Madsen et al., 2021). Sleep quality has been less widely investigated relative to other psychological approaches, however it is important that athletes, coaches, and sports professionals work together to address these challenges and promote the overall health of athletes in contexts such as the COVID-19 pandemic (Nassar et al., 2021).

### Impact of COVID-19 on the mental health of soccer athletes

A growing body of knowledge, analyzed here, indicates that anxiety and depression, as well as acute symptoms are usually mild or absent in this population.

Emerging evidence suggests that a considerable proportion of athletes may exhibit psychological alterations and persistent symptoms that can be potentially detrimental to performance and mental health (Bicalho et al., 2020; Ivarsson et al., 2021), which were not reversed with maintenance and training strategies outside the usual sports environment, suggesting that decisions to return to the game and training should be made with caution (Jansen, 2021).

In a previous review study, it was evidenced that the participation of soccer athletes at the male professional level could provide a certain protective effect for mental disorders or suicide (Morales et al., 2021). However, more research is needed with female athletes and other types of associated sports to corroborate the generalization about mental health aspects.

The evidence base on the mental health and well-being of elite athletes is limited by a dearth of high-quality systematic studies, including intervention trials. Based on current evidence, elite athletes appear to experience a broadly comparable risk of high-prevalence mental disorders relative to the general population. A higher risk for a mental health disorder may be experienced by elite athletes who are injured, approaching/in retirement, or experiencing performance difficulty. Although the importance of the mental health of the elite athlete is gaining greater attention, targeted and specific models of attention for the disorder have yet to be established for this group (Rice et al., 2016; Gouttebarge et al., 2022).

### Strengths, innovations, and practical applications

The present study evaluated psychological aspects in the population of soccer athletes during the COVID-19 pandemic. Articles with this focus were collected from January 2020 to July 2023 and were systematically reviewed according to PRISMA guidelines.

The present review summarizes a robust body of evidence. From the results of the study, it seems appropriate to develop alternatives and support professional athletes during the COVID-19 pandemic. To improve indicators of mental health and overall athlete satisfaction and well-being during and after the pandemic, the development of guidelines related to the monitoring and control of aspects related to athletes’ mental health is recommended.

In the context of soccer, teams of experts working with athletes should pay special attention to alterations in psychological aspects and mental state before and during situations, such as in pandemic times. In the future, more studies with interventions and/or protocols that already have a positive impact on athletes in different modalities, such as *mindfulness*, sleep hygiene, relaxation, for example, should be performed and applied to athletes, without, however, disregarding the methodological limitations of the study designs. In addition, further studies should minimize or exclude potential confounding factors that reduce certainties in the conclusions of studies on this topic.

### Limitations

Although this systematic review provides a comprehensive overview of elite soccer, it has limitations. The articles reviewed in this study were mostly cross-sectional. It was not possible to analyze the prevalence and incidence of the psychological variables investigated, and it was not possible to perform a meta-analysis. Potential factors, such as age variability, competitive level, and athletes’ experience may have hindered the strength of the association between psychological factors and mental health in the studies selected for this review.

Worsening psychological aspects were observed in most of the included studies, but this may not be causally related to COVID-19, given that most control parameters were not presented (e.g., uninfected athletes, infected athletes), including information on athletes with diagnoses of psychological problems prior to the pandemic, and lockdown.

This review also reveals that the absence of control groups or baseline data (i.e., before lockdown) for the assessment of the pandemic phenomenon in the soccer universe are potentially confounding factors (e.g., previous psychological problems of the pandemic, state or trait of anxiety, for example). Inconsistent data or lack of clarity in some included studies are relevant limitations of the literature that should be addressed in future investigations.

Only published articles were included in the review, the gray literature was not included. In addition, the use of an electronic questionnaire as a research tool may have influenced the response bias and the systematic bias given the rigor in controlling the context during the evaluations.

Despite all the limitations presented, it should be taken into account that the understanding of every phenomenon related to the COVID-19 pandemic in the sports universe is still recent and despite this it was possible to gather the information in a rigorous way, as presented in this review.

## Conclusion

The results demonstrate the negative impact of the COVID-19 pandemic on the psychological aspects and mental health of elite soccer athletes during the emergency phase of the pandemic in the year 2020. Because it is a recent topic, most studies used observational methods. Among the most commonly observed effects were increased levels of anxiety, depression, and worsening mental well-being in elite soccer athletes. Studies have shown important mood swings related to athletes’ characteristics and maintaining fitness during the pandemic. For other variables, such as coping, resilience, and monitoring of sleep quality, although there are a reduced number of studies, positive results were shown for soccer athletes. Studies with better methodological designs with controlled experimental interventions are recommended in the future to mitigate the negative effects of the pandemic for this population of athletes.

It is crucial to acknowledge that athletes, especially soccer players, have faced unique challenges during the pandemic, such as disruptions in regular training, postponed competitions, and the need to adhere to strict health protocols. These adversities can have significant impacts on players’ performance and well-being, both in the short and long term.

Therefore, we urge researchers to carefully consider the design of their studies, ensuring that their methodological approaches are robust and comprehensive. This includes collecting data from players before, during, and after the pandemic to properly assess the full impact of these unusual circumstances.

Furthermore, the implementation of controlled experimental interventions is essential to identify effective strategies to support players, such as tailored fitness programs, psychological support, and stress management techniques. These interventions can help mitigate the negative effects of the pandemic and promote the well-being of players.

## Data availability statement

The original contributions presented in the study are included in the article/supplementary material, further inquiries can be directed to the corresponding author.

## Author contributions

AA: Conceptualization, Data curation, Formal analysis, Funding acquisition, Methodology, Project administration, Resources, Validation, Visualization, Writing – original draft, Writing – review & editing. AD’O: Data curation, Investigation, Methodology, Writing – original draft, Writing – review & editing. HN: Formal analysis, Funding acquisition, Methodology, Resources, Writing – review & editing. GG: Formal analysis, Validation, Writing – original draft. WC: Conceptualization, Data curation, Formal analysis, Investigation, Methodology, Project administration, Supervision, Validation, Visualization, Writing – original draft, Writing – review & editing.
